# IGF2 improves the developmental competency and meiotic structure of oocytes from aged mice

**DOI:** 10.18632/aging.202214

**Published:** 2020-12-09

**Authors:** Tahir Muhammad, Yanling Wan, Qianqian Sha, Jianfeng Wang, Tao Huang, Yongzhi Cao, Mengjing Li, Xiaochen Yu, Yingying Yin, Wai Yee Chan, Zi-Jiang Chen, Li You, Gang Lu, Hongbin Liu

**Affiliations:** 1 Center for Reproductive Medicine, Cheeloo College of Medicine, Shandong University, Jinan 250012, Shandong, China; 2 Key Laboratory of Reproductive Endocrinology of Ministry of Education, Shandong University, Jinan 250012, Shandong, China; 3 Shandong Key Laboratory of Reproductive Medicine, Jinan 250012, Shandong, China; 4 Shandong Provincial Clinical Research Center for Reproductive Health, Jinan 250012, Shandong, China; 5 National Research Center for Assisted Reproductive Technology and Reproductive Genetics, Shandong University, Jinan 250012, Shandong, China; 6 Fertility Preservation Laboratory, Reproductive Medicine Center, Guangdong Second Provincial General Hospital, Guangzhou 510317, China; 7 CUHK-SDU Joint Laboratory on Reproductive Genetics, School of Biomedical Sciences, The Chinese University of Hong Kong, Hong Kong 999077, China; 8 Shanghai Key Laboratory for Assisted Reproduction and Reproductive Genetics, Shanghai 200000, China; 9 Center for Reproductive Medicine, Ren Ji Hospital, School of Medicine, Shanghai Jiao Tong University, Shanghai 200135, China

**Keywords:** maternal age, IGF2, meiosis, mitochondria, oocyte quality

## Abstract

Advanced maternal-age is a major factor adversely affecting oocyte quality, consequently worsening pregnancy outcomes. Thus, developing strategies to reduce the developmental defects associated with advanced maternal-age would benefit older mothers. Multiple growth factors involved in female fertility have been extensively studied; however, the age-related impacts of various growth factors remain poorly studied. In the present study, we identified that levels of insulin-like growth factor 2 (IGF2) are significantly reduced in the serum and oocytes of aged mice. We found that adding IGF2 in culture medium promotes oocyte maturation and significantly increases the proportion of blastocysts: from 41% in the untreated control group to 64% (50 nM IGF2) in aged mice (*p* < 0.05). Additionally, IGF2 supplementation of the culture medium reduced reactive oxygen species production and the incidence of spindle/chromosome defects. IGF2 increases mitochondrial functional activity in oocytes from aged mice: we detected increased ATP levels, elevated fluorescence intensity of mitochondria, higher mitochondrial membrane potentials, and increased overall protein synthesis, as well as increased autophagy activity and decreased apoptosis. Collectively, our findings demonstrate that IGF2 supplementation in culture media improves oocyte developmental competence and reduces meiotic structure defects in oocytes from aged mice.

## INTRODUCTION

Oocyte quality, an indicator of female fertility, is essential for early embryonic developmental competency and pregnancy outcomes. Among known factors correlated with oocytes quality, advanced maternal age is understood as a major deleterious factor which is accompanied by declining oocyte quality [1–3]. Age-related decline in oocyte quality is associated with a range of defects, including reduced oocyte meiotic maturation, mitochondrial dysfunction, impaired spindle assembly and chromosomes misalignment in oocytes [4, 5]. Mitochondria, as indispensable contributors to cellular energy metabolism in oocytes, are necessary for cellular calcium homeostasis, meiosis, regulation of apoptosis, and cellular translation during oocyte and embryo development [6–8]. Mitochondrial dysfunction in oocytes due to advanced maternal age contributes to higher chromosomal abnormalities, elevated ROS production, and ultimately failure of molecular and cellular process which lead to infertility [9, 10]. Such defects during meiosis promote the chances of infertility, miscarriage, and congenital malformation. Thus, it should be useful to elucidate the changes in cellular functions of compromised oocytes that occur with advanced age, potentially thereby helping to develop strategies for reducing these defects. Such knowledge would very likely have a significant impact on improving the success rates of assisted reproductive technologies (ART).

Growth factors are ligands that interact with specific receptors and regulate signaling cascades. A number of growth factors that are involved in female fertility have been extensively studied [11]; however, the age-related impacts of various growth factors remain poorly studied. Previous studies have shown that different growth stimulants essential for early embryonic development and implantation success are secreted from the female reproductive tract [12, 13]. Different growth factors are known to confer beneficial effects for oocyte and embryo developmental competency when added in culture medium [14]. Among known reproduction-related growth factors of the insulin-like growth factors (IGFs) family, insulin-like growth factor 2 (IGF2) is particularly well-studied; it is highly expressed in granulosa cells, follicles, oocytes, and embryos of diverse mammalian species [15–18], and is understood as an essential regulator of the human ovarian system [16].

IGF2 is a highly conserved 67-amino acid, single chain secreted protein with multiple known physiological functions affecting female fertility [19]. Previous work has established functional roles for IGF2 in processes including follicular growth, oocyte and embryo development, reducing placental apoptosis and increasing fetal growth [20–23]. In addition, our recently published experimental finding and other clinical studies have demonstrated a functional impact for IGF2 on human and mouse embryo development [20, 24, 25]. Furthermore, recent reports about adult neuronal culture-derived cell lines have also demonstrated that IGF2 increases mitochondrial functional activity by reducing oxidative stress, as well as by increasing immunofluorescence staining intensity for mitochondria and increasing mitochondrial membrane potential [26, 27]. Despite these numerous basic studies, the potential application of IGF2 is relatively unexplored, so little is known at the cellular and molecular level about how manipulation of IGF2 levels in for example oocytes from aged females may confer improvements in embryonic growth or even overall pregnancy outcomes.

Here, after detecting significant reductions in IGF2 levels in the serum and oocytes of aged mice, we observed increased developmental competency and reduced meiotic defects in oocytes from aged mice after adding IGF2 in the culture medium. Our findings strongly support the application potential of IGF2 for helping to overcome age-related meiotic structural developmental defects. IGF2 can potentially help to improve the currently standard culturing conditions used for assisted reproduction technologies.

## RESULTS

### Aged mice have reduced serum IGF2 protein levels and their oocytes have reduced *Igf2* expression

**Figure 1:**
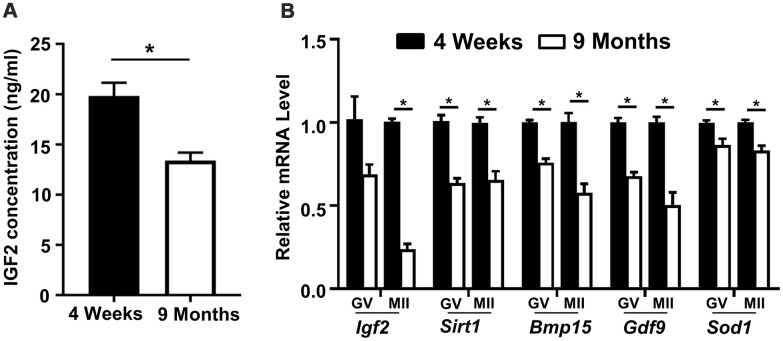
**Reduced serum IGF2 protein levels and reduced *Igf2* expressions in oocytes from aged mice.** (**A**) Serum IGF2 concentration in young and aged mice assessed via ELISA. n=3 for each group. (**B**) qPCR results showing mRNA levels of *Igf2* and target genes in GV-stage and MII-stage oocytes from young and aged mice. Student’s *t-*test (two-tailed). *p < 0.05. Error bars indicate the SEM.

In light of previous reports of fertility-promoting roles for IGF2, we investigated the potential involvement of this growth factor in oocyte development in aged mice of 9 months. We first evaluated the IGF2 level in blood sera samples from young (4 weeks) and aged (9 months) mice using ELISA, which revealed that the aged mice had significantly reduced IGF2 concentrations (Figure 1A). Further associating an age-related decline in IGF2 levels with age-related declines in fertility, a qPCR analysis of GV-stage and MII-stage oocytes retrieved from young and aged mice also revealed reductions in the mRNA levels of *Igf2* [28] (Figure 1B). Further, we detected significant reductions in the levels of known antioxidant and oocyte-specific genes, including *Sirt1, Bmp15, Gdf9*, and *Sod1* (Figure 1B). Collectively, these findings suggest that reduced IGF2 levels may be involved in the impaired oocyte development known to occur in aged mice.

### Treatment of oocytes from aged mice with IGF2 improves meiotic maturation and early embryonic development

**Figure 2:**
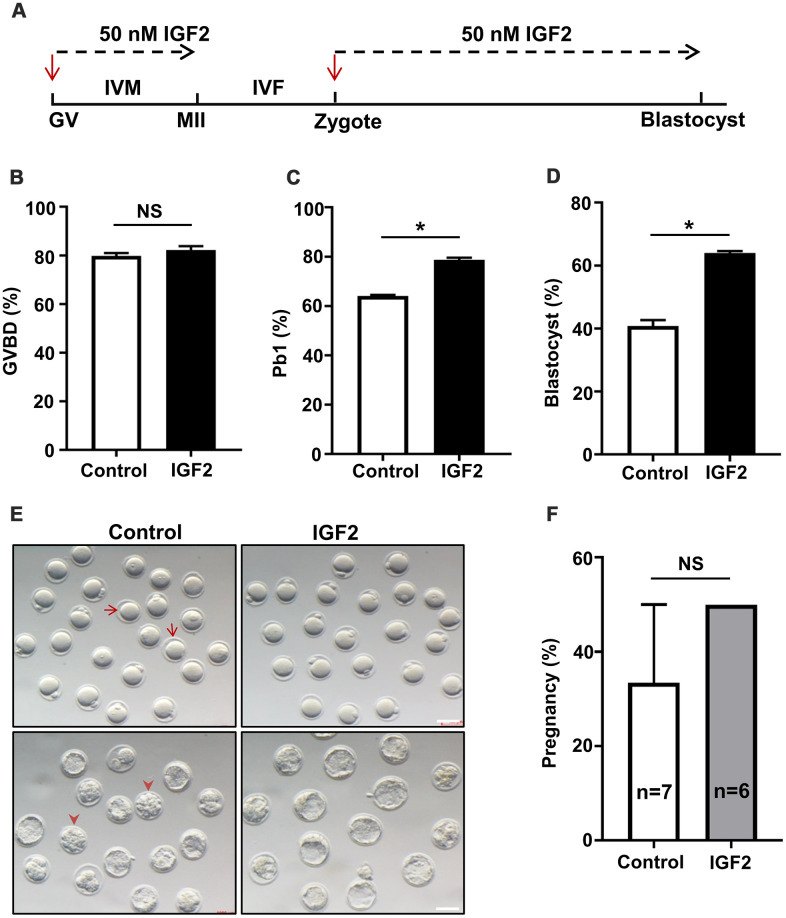
**IGF2 administration in culture medium improves the oocytes maturation and early embryonic developmental competence of aged mice.** (**A**) Schematic diagram showing IGF2-treatment of oocytes and early embryos in M16 medium *in vitro*. (**B, C**) Quantitative analysis of GVBD (**B**) and Pb1 extrusion in control oocytes (n = 164) and IGF2-treated oocytes (n = 180) (**C**). (**D**) Quantitative analysis of blastocysts in control embryos (n = 218) and IGF2-treated embryos (n = 222). (**E**) Morphology of *in vitro* cultured oocytes and embryos examined for development within specific time frames. Arrows indicate the oocytes which failed to extrude a polar body; arrowheads denote embryos which failed to develop into blastocysts. Scale bar, 100 μm. (**F**) Quantitative analysis of the pregnancy rate in the control and IGF2-treated embryos. 15 blastocysts were transferred into the uterus of each female. n here indicates the numbers of females used as recipients. *p < 0.05. A Student’s *t-*test (two-tailed). NS, not significant.

Previous studies have shown that adding IGF2 to cultured medium increases the meiotic maturation of porcine oocytes [22]. To investigate whether IGF2 supplementation in culture media functionally impacts oocytes development in aged mice, GV-stage oocytes were collected from aged mice and cultured in medium with or without 50 nM IGF2 (Figure 2A). We observed that the presence of IGF2 had no effect on meiotic resumption; as no difference in the percentage of germinal vesicle breakdown (GVBD) was noticed after 3 h of *in vitro* culture (Figure 2B). However, IGF2 increased the polar body (Pb1) extrusion rate significantly (*p* < 0.05) (Figure 2C–2E). We observed a significant increase in oocyte maturation in the presence of IGF2: whereas a majority of the control oocytes arrested at the GVBD-stage, more than 79% percent of the IGF2-exposed oocytes proceeded into the MII-stage (Figure 2C–2E).

We additionally explored potential functional impacts of IGF2 on embryonic development by culturing zygotes from aged mice in M16 medium with or without 50 nM IGF2. The presence of IGF2 in the culture medium increases the proportion of zygotes that developed into blastocysts: from 41% in the untreated control group to 64% in the IGF2 group (*p* < 0.05) (Figure 2D, 2E). Note that most of the embryos in control group arrested at the compact morula-stage (Figure 2E). We also examined developmental-fate-related effects of IGF2-treatment *in vivo* with an embryo transfer experiment which showed that pregnancy rates did not differ between control and IGF2-treated embryos (Figure 2F). These results suggest that IGF2 does not apparently enhance embryonic development *in vivo*. Thus, our data suggest that IGF2 may have the potential to improve the meiotic maturation and early embryonic developmental competency of oocytes from aged mice.

### IGF2 promotes the spindle assembly and chromosome alignment while also reducing ROS levels in aged mouse oocytes

**Figure 3:**
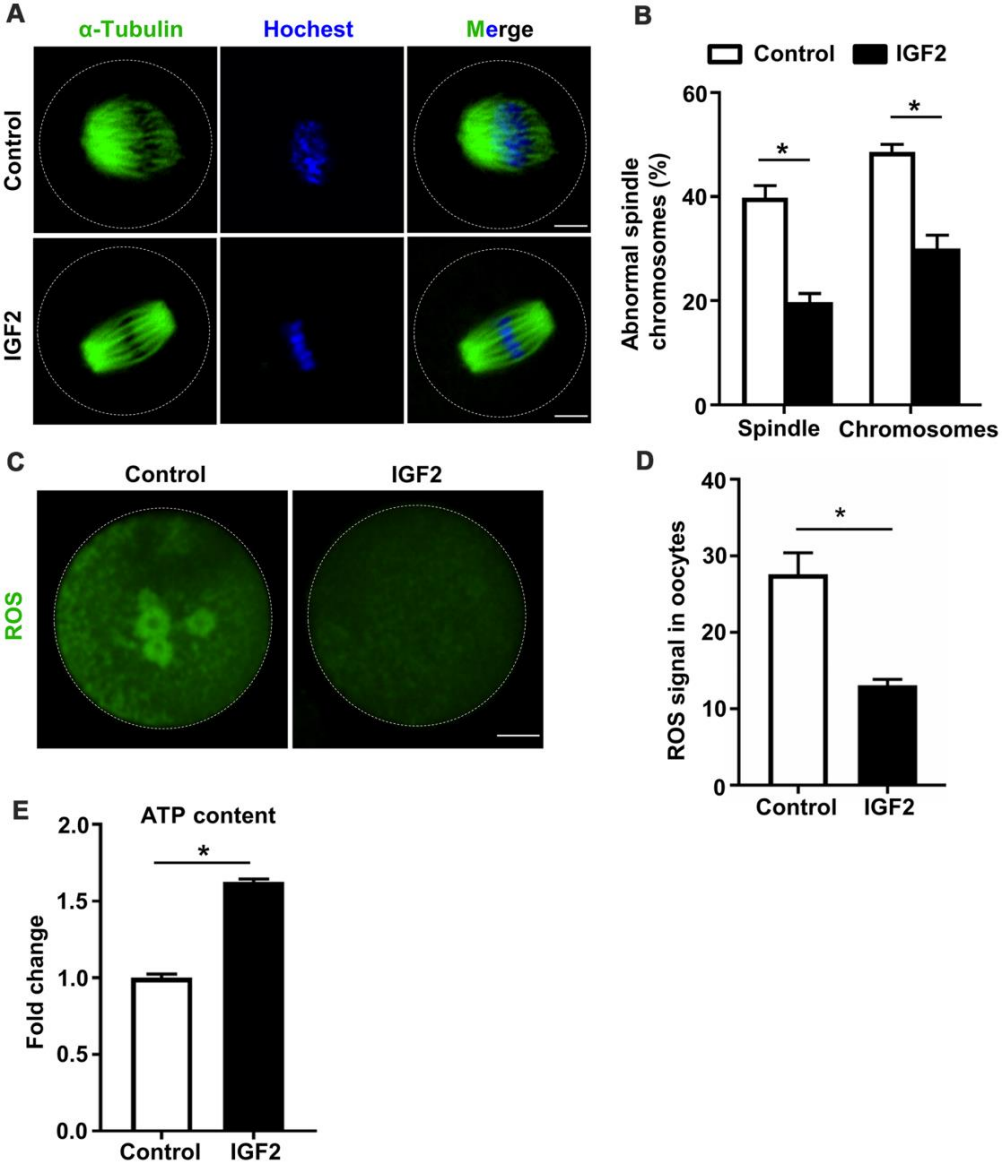
**IGF2 ameliorates the meiotic defects of aged mouse oocytes.** (**A**) Representative images of spindle/chromosome organization in control and IGF2-treated oocytes from aged mice. Spindles were stained with an antibody against *α*-tubulin (green), and chromosomes were counter-stained with Hoechst 33342 (blue). Scale bar = 30 μm. (**B**) Quantification of abnormal spindle/chromosomes oocytes in control (n = 95) and IGF2-treated (n =105) oocytes groups. A Student’s *t-*test (two-tailed). *p < 0.05. Error bars indicate the SEM. (**C**) Representative images of CM-H2DCFDA fluorescence (green) in control and IGF2-treated oocytes. Scale bar = 20 μm. (**D**) Quantification of ROS signals in control oocytes (n = 25) and IGF2-treated oocytes (n = 21). A Student’s *t-*test (two-tailed). *p < 0.05. Error bars indicate the SEM. (**E**) Adenosine triphosphate (ATP) contents in control oocytes (n = 50) and IGF2-treated oocytes (n = 50). A Student’s *t-*test (two-tailed). *p < 0.05. Error bars indicate the SEM.

Previous studies have established that oocytes quality is influenced by multiple factors including spindle morphology, chromosome alignment, mitochondrial activity, and studies of aged mouse oocytes have reported aberrantly high frequencies of spindle and chromosomal abnormalities [29]. We investigated whether administration of IGF2 during *in vitro* culture could improve the quality of oocytes from aged mice. Specifically, we retrieved immature GV-stage oocytes from aged mice and cultured them in M16 medium with or without 50 nM IGF2 until MII-stage. Immunofluorescence analysis of MII-stage oocytes revealed that the IGF2 treatment resulted in a significant reduction in both spindle and chromosomal alignment abnormalities (Figure 3A, 3B). We found that the majority of the IGF2-treated oocytes displayed typical barrel-shaped spindles with well-aligned chromosomes (Figure 3A). In addition, we found that the ROS level was significantly reduced in the IGF2-treated oocytes compared to controls (Figure 3C, 3D) and also detected significantly increased ATP content in the IGF2-treated oocytes (Figure 3E). Collectively, these *in vitro* results show that IGF2 can improve the quality of oocytes from aged mice, specifically by promoting spindle assembly and chromosomes alignment and by reducing ROS levels.

### IGF2 improves mitochondrial function in oocytes from aged mice

**Figure 4:**
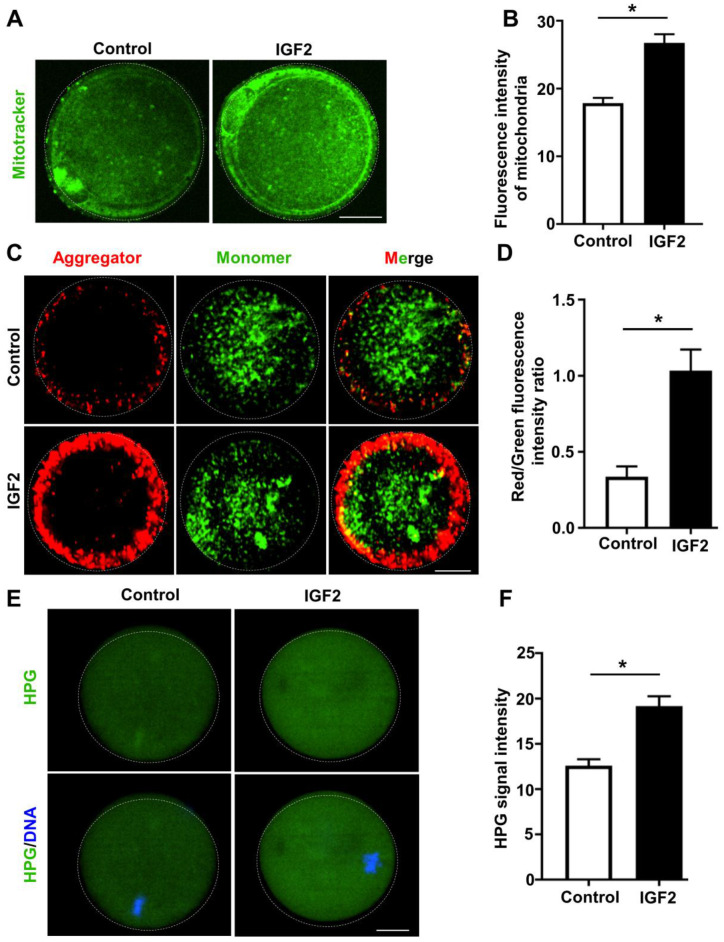
**IGF2 improves the mitochondrial functional activity of oocytes from aged mice.** (**A**) Mitochondria were stained with mitotracker Green FM (green). Scale bar = 20 μm. (**B**) Quantification of mitochondrial distribution signals in control oocytes (n = 26) and IGF2-treated oocytes (n = 25). A Student’s *t-*test (two-tailed). *p < 0.05. Error bars indicate the SEM. (**C**) JC-1 staining showing the mitochondrial membrane potential (MMP) in control and IGF2-treated oocytes. (**D**) Quantification of the red/green fluorescence intensity ratio in control oocytes (n = 40) and IGF2-treated oocytes (n = 35). A Student’s *t-*test (two-tailed). *p < 0.05. Error bars indicate the SEM. (**E**) HPG Fluorescent staining showing total protein synthesis in MII-stage oocytes with or without IGF2-treatment. Oocytes were incubated in M16 medium with 50 μM HPG for 1 h prior to staining. Scale bar = 30 μm. (**F**) Quantification of HPG signal intensity in control (n = 28) and IGF2-treated (n = 29) oocytes. *p < 0.05. A Student’s *t-*test (two-tailed). Error bars indicate the SEM.

Recall that mitochondrial activity is known to be indicative of oocytes quality [30]; previous work with oocytes from aged mice has revealed highly defective mitochondrial function, including defects in mitochondrial distribution and reduced mitochondrial membrane potential (MMP) [31]. We examined the impacts of IGF2 on mitochondrial function in oocytes from aged mice with experiments wherein *in vitro*-matured MII-stage oocytes were cultured with or without IGF2. Immunofluorescence analysis revealed that IGF2 treatment resulted in significantly increased immunofluorescence staining intensity for mitochondria: higher fluorescence intensity of Mitotacker Green FM was observed in IGF2-treated oocytes compared to un-treated control oocytes (Figure 4A, 4B). Moreover, JC-1 staining assays revealed that treatment of aged mouse oocytes with IGF2 increased the MMPs index (Figure 4C, 4D), clearly indicating a role for IGF2 in somehow promoting mitochondrial function in aged oocytes.

Previous work has shown that protein metabolism is highly defective in aged mouse oocytes [32]. To test whether IGF2 administration could improve global protein synthesis in oocytes from aged mice, control and IGF2-treated MII-stage oocytes were incubated in a medium containing L-homopropargylglycine (HPG, a methionine analogue that is incorporated into nascent proteins during active protein synthesis) for 1 h at 37° C. HPG signals are indicative of overall translational activity [33], and our data revealed that administration of IGF2 in culture medium could improve the translation activity in oocytes from aged mice: increased HPG signal intensity was detected in IGF2-treated oocytes relative to control oocytes (Figure 4E, 4F). Taken together, these results suggest that administration of IGF2 can activate mitochondrial function in a way that consequently improves the quality of oocytes from aged mice.

### IGF2 improves the ultrastructure of mitochondria of oocytes from aged mice

**Figure 5:**
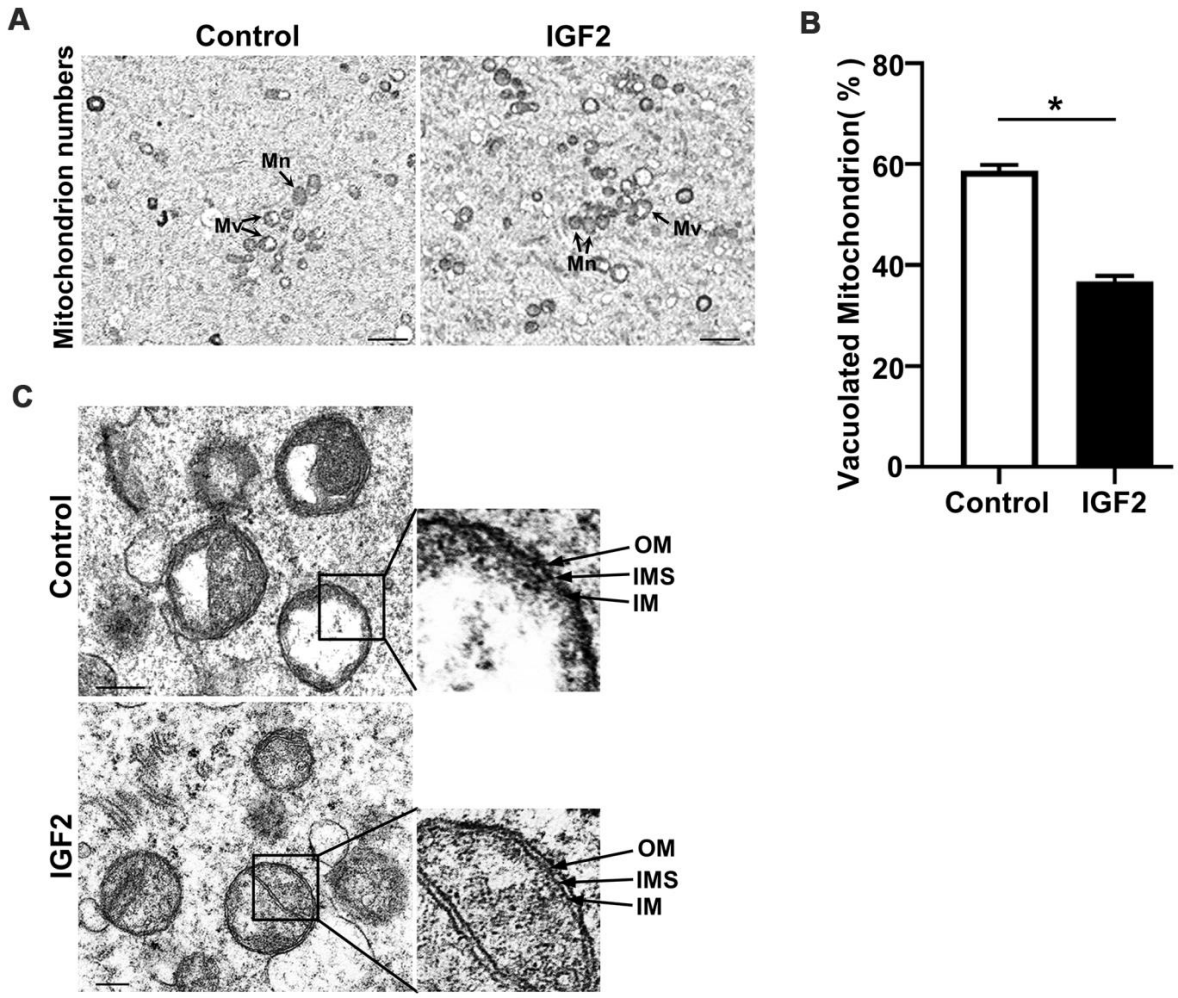
**IGF2 improves the mitochondrial ultrastructure of oocytes from aged mice.** (**A**) Representative TEM micrographs of mitochondria from control and IGF2-treated oocytes at 2,500x magnification. Scale bar = 1 μm. Note the normal (Mn) and vacuolated (Mv) mitochondria. (**B**) Quantification of mitochondria per defined region of interest (ROI) in control and IGF2-treated oocytes. n=9 oocytes for each group. A Student’s *t-*test (two-tailed). *p < 0.05. Error bars indicate the SEM. (**C**) Representative TEM micrographs of mitochondria from control and IGF2-treated oocytes at 60,000x magnification. Inner membrane (IM), outer membrane (OM), and intermembrane space (IMS). Scale bar = 200 nm.

Given our finding that IGF2 administration mediates the functional activity of mitochondria, we next assessed whether IGF2 supplementation exerts any functional impact(s) on the ultrastructure of mitochondria in oocytes from aged mice. Transmission electron microscopy of MII-stage oocytes from aged mice revealed a normal morphology for mitochondria shape, with defined cristae in IGF2-treated oocytes; in contrast many mitochondria in un-treated control oocytes had vacuolated cristae (Figure 5A, 5B). Most IGF2-treated oocytes had mitochondria with clearly visible intact inner membranes, outer membranes, and well-defined intermembrane spaces, whereas un-treated control oocytes contained many ruptured and discontinuous inner and outer membranes with disrupted intermembrane structures (Figure 5C). Thus, IGF2 treatment can improve the ultrastructure of mitochondria in oocytes from aged mice.

### IGF2 promotes the autophagy and also reduces the apoptotic index of oocytes from aged mice

**Figure 6:**
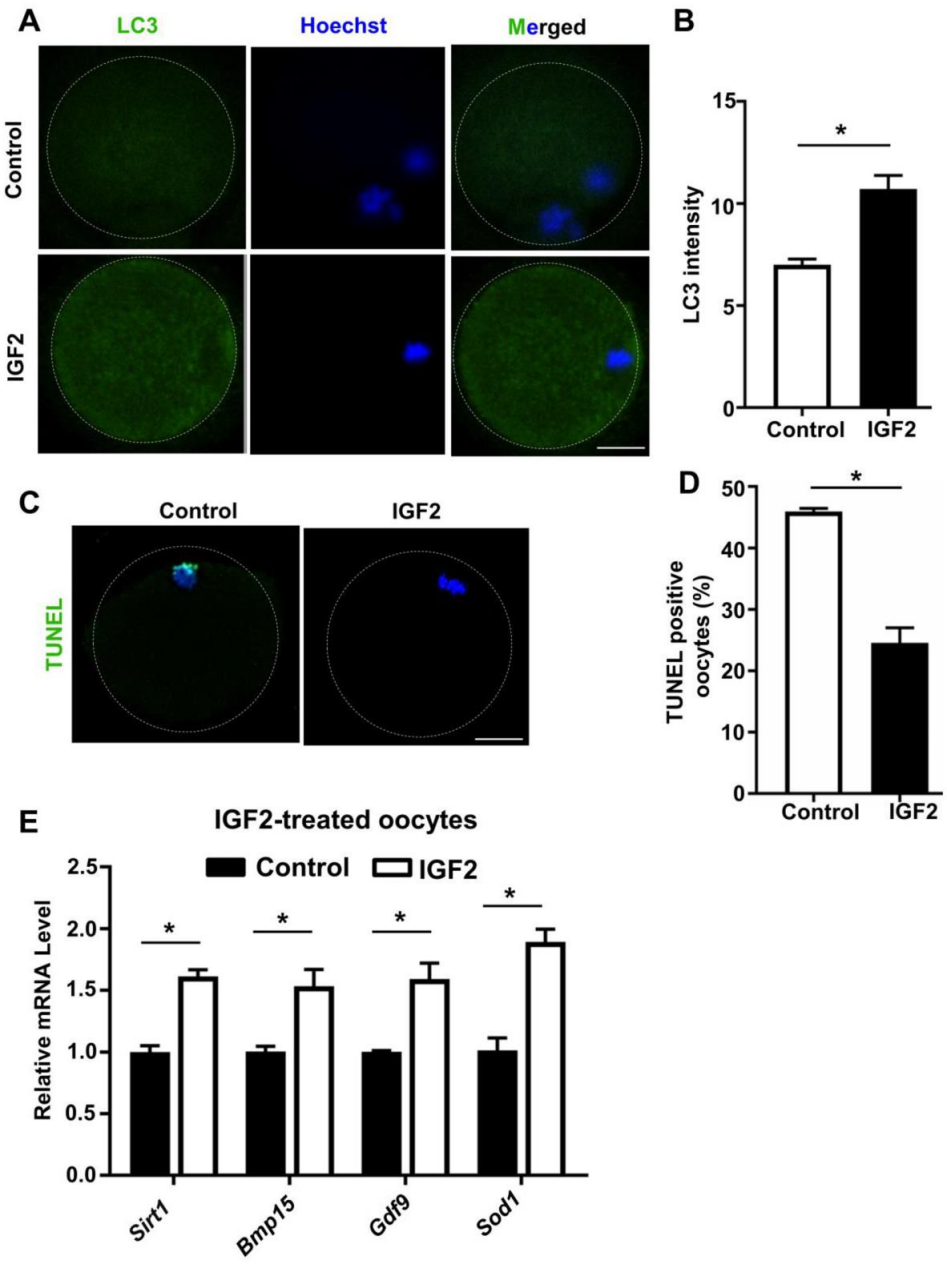
**IGF2 reduces the apoptosis and promotes the level of autophagy in aged mouse oocytes.** (**A**) LC3 staining showing the extent of autophagy occurring in control and IGF2-treated oocytes. (**B**) Quantification of LC3 intensity in control (n = 34) and IGF2-treated oocytes (n = 25). A Student’s *t-*test (two-tailed). *p < 0.05. Error bars indicate the SEM. (**C**) TUNEL assay of control and IGF2-treated oocytes from aged mice. A green fluorescence signal indicates TUNEL-positive oocytes. Apoptotic signals were observed after 16 h of *in vitro* culture. DNA was counterstained with DAPI. Scale bar = 30 μm. (**D**) The percentage of apoptosis-positive oocytes in control (n = 61) and IGF2-treated oocytes group (n = 44). A Student’s *t-*test (two-tailed). *p < 0.05. Error bars indicate the SEM. (**E**) qPCR results showing mRNA levels of *Sirt1, Bmp15, Gdf9,* and *Sod1* in MII-stage oocytes after *in vitro* maturation with or without IGF2-treatment. *p < 0.05. A Student’s *t-*test (two-tailed). Error bars indicate the SEM.

Autophagy is an essential cellular process that degrades degenerated proteins and cellular organelles to recycle their components in the cytoplasm. Previous reports have shown that increased autophagy can be induced in oocytes from aged cow via supplementation with resveratrol in the culture medium, and such up-regulated autophagy was associated with improved early embryonic development outcomes [34, 35]. We examined whether supplementation with IGF2 may promote autophagy in aged mouse oocytes in experiments using the total LC3 level as an indicator for autophagy activity. The autophagy index of oocytes from aged mice was significantly increased by supplementation with IGF2 in the culture medium compared to controls (Figure 6A, 6B).

A previous study reported that inhibition of autophagy increases apoptosis in porcine oocytes, which consequently reduced oocyte meiotic maturation [36], and IGF2 was shown to reduce apoptosis in cultured BeWO cells [23]. We checked whether IGF2 supplementation of culture medium has any impact(s) on the extent of oocyte apoptosis in aged mice, and found that IGF2-treatment significantly reduced apoptosis compared to controls after 16 h of culturing (Figure 6C, 6D).

Previously, it was shown that increased expressions of sirutin family member (*SIRT1*) and antioxidant relevant genes are indicator of oocytes development in aged mouse oocytes [29, 37]. We found that administration of IGF2 to the culture medium significantly induced the expression of genes including *Sirt1, Bmp15,* *Gdf9*, and *Sod1* in oocytes from aged mice compared to controls (Figure 6E). Overall, these results suggest that IGF2 can maintain the autophagy level and can reduce the apoptotic index of oocytes from aged mice.

## DISCUSSION

The quality of gametes is dictated by cytoplasmic and meiotic competence during oocyte maturation [38]. The decline in oocyte quality associated with advanced maternal-age reduces embryonic developmental competency, which adversely affects female fertility [39]. Currently, ART has been widely used for the treatment of infertile couples; however, the reduced quantity and compromised quality of oocytes from aged women is still a pressing challenge facing patients, clinicians, and embryologists. Thus, developing strategies to reduce age-related developmental and organizational defects in oocytes would benefit older mothers.

The functional activity and involvement of IGF2 in folliculogenesis and in the development of oocytes, embryos, fetuses, and placenta has been established in previous studies [20, 22, 40, 41]. However, to our knowledge ours is the initial study examining impacts of IGF2 supplementation on the development of oocytes from aged mice, and our finding that IGF2 can positively impact the organization of meiotic structures is highly promising. We found reduced IGF2 levels in serum and decreased *Igf2* mRNA expression in oocytes of aged mice, consistent with previous reports about reduced serum IGF2 levels upon aging and a decline in *Igf2* mRNA expression in an animal stress model [42–44]. Our experiments indicated that IGF2 supplementation of culture media improved the *in vitro* development of oocytes from aged mice, assessed in terms of both meiotic maturation and blastocyst formation. Note that the choice of the 50nM IGF2 concentration was based on dose-depended trials in our previously published work [20]. Previous studies have reported potential functional impacts of IGF2 for increasing embryonic developmental competency in mice and humans [20, 25]. Previously, IGF2 supplementation in culture medium was shown to improve the meiotic maturation of porcine oocytes [22]. Clinically, the IGF2 level in human follicular fluid has also been used to assess the developmental capacity of human oocytes, suggesting that IGF2 may be a useful biomarker of meiotic resumption [24]. These studies support the notion of an oocyte and embryo growth-promoting potential for IGF2 in the culture medium. Previously, it was shown that the growth-promoting activity of IGF2 is mediated by its receptors (IGF-1R, IGF-2R, INSR); IGF2 binds to these specific receptors, which induces phosphorylation and subsequent activation of the PI3K/Akt signaling pathway and promotes embryo development and cell proliferation [45–48].

Mitochondria generate ATP via oxidative metabolism, and mitochondrial activity can be used to assess the quality of oocytes. Previous studies have established requirements for a low ROS index and for a relatively high ATP level for proper spindle assembly and chromosome alignment in oocytes; both ROS and ATP metabolism are directly associated with mitochondrial functional responses [49, 50]. Advanced maternal-age is known to adversely affect mitochondrial function in oocytes, and there are reports that aged oocytes exhibit increased abnormalities in spindle and chromosome organization, elevated ROS indices, and reduced MMP values [51, 52]. Our results indicated that IGF2 supplementation of culture media can help to minimize these meiotic defects. A previous report in adult neuronal culture-derived cells showed that IGF2 supplementation can reduce oxidative damage and promote mitochondrial activity, resulting in an overall improvement in the functional activity of mitochondria and increased MMP [26].

Previous studies have shown that cellular translation machinery is essential for maintaining normal spindle morphology and chromosome alignment during the meiotic maturation of oocytes, and this machinery is also required for early embryonic development [53, 54]. We know that translational machinery in aged mouse oocytes is highly disturbed, and this has been assumed to confer profound deleterious impacts on the development of gametes [32]. Our results show that translational activity can be increased in oocytes from aged mice after adding IGF2 to the culture media. A previous study of an embryonal carcinoma cell line reported that IGF2 activates translation initiation [55]. It is known that autophagy is essential for proper meiotic maturation of porcine oocytes, and the reduced autophagy levels that characterize oocytes from aged mice lead to diverse meiotic defects [36, 56]. Our results are consistent with previous work reporting a functional impact of IGF2 in maintaining relatively high autophagy levels in osteosarcoma cells (which induced an autophagic state of dormancy that protects cells against stress) [57, 58]. The reduced autophagy and increased mitochondrial dysfunction that occurs during the maturation of oocytes activate the mitochondrial-related apoptotic signaling pathway [36, 59]. Previous reports have shown the increased apoptotic level in oocytes from aged mice and human consequently impairs meiotic maturation and causes embryonic developmental defects [60–62]. Previous studies have shown that IGF2 triggers anti-apoptotic signaling pathways in human trophoblast cells and also in mice placental cells [23, 63], findings consistent with our observation of reduced apoptosis in oocytes from aged mice that were cultured in media supplemented with IGF2.

In conclusion, our study indicates that IGF2 promotes the developmental competence of oocytes from aged mice and may specifically impact the meiotic structures in these oocytes. Our work confirms age-related decreases in IGF2 levels and clearly highlights the strong clinical promise for deploying IGF2 in ART to reduce age-related meiotic developmental defects. Given reports of IGF2 deficits in non-aged female infertility patients (*e.g.*, oocytes from obese women), perhaps IGF2 supplementation in *in vitro* culture systems could improve the yield of quality embryos derived from obese women, which should also benefit implantation success and improve overall pregnancy outcomes. Notably, our ongoing initial trials of IGF2 supplementation of media for culturing oocytes from obese mice is also indicating that IGF2 can improve developmental efficiency of oocytes and early embryos. Further investigations regarding the potential application of IGF2 for ART of oocytes from aged women, including assessment of pregnancy outcomes and safety evaluations, are warranted; these will be needed to assess the feasibility and safety of any IGF2-based clinical interventions.

## MATERIALS AND METHODS

### Mice

Young (4 weeks) and aged (42–45 weeks old) ICR female mice (Charles River Laboratories China Inc) were selected for this experiment. All animal experimental protocol was performed accordance to the ethical guidelines approved by the Animal Care and Research Committee of Shandong University.

### Oocytes collection and culture

To get fully grown GV-stage oocytes, aged mice were superstimulated with 5 IU pregnant mare’s serum gonadotropin (PMSG) injection. After 48 h of PMSG injection, cumulus oocytes complex were obtained by manually rupturing the ovarian follicles structure. The oocytes were collected and randomly divided into two groups. Oocytes with or without 50 nM IGF2 (100-12, Peprotech), were cultured in the small drops of M16 (M7292; Sigma-Aldrich), and maintained in 5% CO_2_ at 37° C. For collection of MII-stage oocytes, mice received an injection of 5 IU PMSG followed by 5 IU human chorionic gonadotrophin (hCG) after 44 h. MII-stage oocytes were collected after 16 h of hCG and used for *in vitro* fertilization (IVF) experiment.

### Zygotes culture and embryo transfer

MII-stage oocytes were collected and IVF experiment was performed by using sperms from wild-type (WT) male. Zygotes were cultured in M16 medium with or without 50 nM IGF2, and incubated at 37° C in 5% CO2 for observing their embryonic developmental competence. Embryos development and morphology were examined with a stereomicroscope (Nikon SMZ1500). In an experiment related to embryo transfer, blastocysts obtained with or without IGF2-treatment were transferred. WT female mice were used as the recipients (15 embryos were transferred to the uterus of each mouse), and pregnancy rates to term were recorded.

### Estimation of serum IGF2 concentration

The concentration of IGF2 was measured in mouse serum samples by following the manufacturer’s instructions using ELISA kit (RnD system, MG200). Briefly, blood from young and aged mice were collected and put at room temperature for 1 h. Samples were centrifuged at 3000×g for 10 min at 4° C. Serum was collected and stored at -80° C for subsequent assay. The IGF2 concentration was determined in triplicate. The standard curves were generated, and the IGF2 content was calculated using the formula derived from the standard curve.

### RNA extraction and qRT-PCR validation

Total RNA was extracted using RNeasy mini kit (Qiagen) following the manufacturer’s instructions. Genomic DNA (gDNA) was eliminated by digesting with RNase-free genomic DNA eraser buffer (Qiagen), and cDNA was obtained by reverse transcription of RNA using PrimeScript^TM^ reverse transcriptase (Takara). Power SYBR Green Master Mix (Takara) was used on a Roche 480 PCR system for qRT-PCR analysis. The qRT-PCR reactions were performed in triplicate for gene specific primers. The mRNA level was calculated by normalizing to the endogenous mRNA level of actin (internal control) using Microsoft Excel. Primer sequences are shown (Supplementary Table 1, Supporting Information).

### Immunofluorescence

To detect relevant protein, the oocytes were fixed in 4% paraformaldehyde for 30 min, permeabilized with 0.3% Triton X-100 for 20 min. After washing three times, the oocytes were blocked in blocking buffer in PBS with 1% BSA. Oocytes were incubated with a fluorescein isothiocyanate (FITC)-conjugated anti-mouse Alpha tubulin (1:200 dilution, Sigma) antibody, anti-γ-H2AX (1:300 dilution, Abcam), anti-apoptotic (1:1000 dilution, Abcam), and anti-LC3 (1:300, Abcam) for 1 h at room temperature. After washing three times, oocytes were incubated with respective secondary antibodies. DNA was counterstained with DAPI (Sigma) for 10 min at room temperature. Oocytes were washed and mounted on the glass slides and observed under confocal laser microscope (Zeiss LSM 780, Carl Zeiss AG, Germany).

### Determination of ATP levels

The measurement of total ATP content of MII-stage oocytes obtained with and without IGF2-treatment was performed by using ATP testing assay kit (Beyotime). Briefly, 50 oocytes were added to lysis buffer and centrifuged at 12000×g for 10 min. Supernatant was mixed with testing buffer, and ATP concentrations were measured on a luminescence detector (EnSpire Multimode Plate Reader). A 6-point standard curve was generated ranging from 0.01 mM to 1 m and total ATP contents were calculated.

### ROS evaluation

ROS was measured in MII-stage oocytes by using ROS assay kit (Beyotime) by following manufacturer’s instructions. Briefly, control and IGF2-treated oocytes were incubated with 10 μM, 2’,7’ dichlorofluorescein diacetate (DCFH-DA) in M16 medium at 37° C in 5% CO_2_ for 30 minutes. After three washes, oocytes were mounted on glass slides, and examined under confocal laser microscope (Zeiss LSM 780, Carl Zeiss AG, Germany).

### Detection of mitochondrial distribution and JC-1 assay

To detect mitochondrial distribution, MII-stage oocytes were incubated with 400 nmol/L Mito tracker Green FM (Invitrogen) diluted in PBS for 30 minutes at 37° C and fixed in 2% paraformaldehyde for 20 minutes. To evaluate the mitochondrial membrane potential, the oocytes were incubated in M16 culture medium containing 10μM JC-1 (Beyotime Institute of Biotechnology) at 37° C for 30 min. After washing three times in PBS, the oocytes were mounted on glass slides and observed immediately (Zeiss LSM 780, Carl Zeiss AG, Germany). The red and green fluorescents intensities were determined and mitochondrial membrane potential was calculated as the ratio of red and green fluorescent pixels.

### Detection of protein synthesis

The protein synthesis assay was performed as described previously [33] using the Click-iT protein synthesis assay kit (C10428, Life Technologies) following the manufacturer’s instructions. Briefly, the MII-stage oocytes were incubated in culture medium supplemented with 50 μM HPG at 37° C with 5% CO2 for 1 h. Oocytes were fixed with 3.7% formaldehyde followed by permeabilization with 0.5% Triton X-100 for 20 min at room temperature. The HPG signal is indicative of the overall level of translation in oocytes.

### Electron microscope

Electron microscopy (EM) was performed as described previously [64]. Briefly, MII-stage oocytes treated with or without IGF2 were collected, visualized and captured with a transmission electron microscope (TEM, JEOL). The numbers of normal and vacuolated mitochondria were quantified in defined region of interests (ROIs) in the oocyte cytoplasm using IMAGE J (National Institutes of Health, MD, USA).

### Statistical analysis

Data are presented as mean ± SEM of three independent experiments/samples unless otherwise specified. Group comparisons were made by two-tailed unpaired Student’s *t*-tests. *p < 0.05; **P < 0.01, and ***P < 0.001. All analyses were performed using the GraphPad Prism 7 (GraphPad Software, San Diego, CA, USA).

## Supplementary Material

Supplementary Table 1

## References

[1] Pellestor F, Andréo B, Arnal F, Humeau C, Demaille J. Maternal aging and chromosomal abnormalities: new data drawn from in vitro unfertilized human oocytes. Hum Genet. 2003; 112:195–203. 10.1007/s00439-002-0852-x 12522562

[2] Christopikou D, Tsorva E, Economou K, Shelley P, Davies S, Mastrominas M, Handyside AH. Polar body analysis by array comparative genomic hybridization accurately predicts aneuploidies of maternal meiotic origin in cleavage stage embryos of women of advanced maternal age. Hum Reprod. 2013; 28:1426–34. 10.1093/humrep/det053 23477909

[3] Yamamoto T, Iwata H, Goto H, Shiratuki S, Tanaka H, Monji Y, Kuwayama T. Effect of maternal age on the developmental competence and progression of nuclear maturation in bovine oocytes. Mol Reprod Dev. 2010; 77:595–604. 10.1002/mrd.21188 20575084

[4] Liu XJ. Targeting oocyte maturation to improve fertility in older women. Cell Tissue Res. 2016; 363:57–68. 10.1007/s00441-015-2264-y 26329301

[5] Eichenlaub-Ritter U, Vogt E, Yin H, Gosden R. Spindles, mitochondria and redox potential in ageing oocytes. Reprod Biomed Online. 2004; 8:45–58. 10.1016/s1472-6483(10)60497-x 14759287

[6] Brevini TA, Vassena R, Francisci C, Gandolfi F. Role of adenosine triphosphate, active mitochondria, and microtubules in the acquisition of developmental competence of parthenogenetically activated pig oocytes. Biol Reprod. 2005; 72:1218–23. 10.1095/biolreprod.104.038141 15659704

[7] De los Reyes M, Palomino J, Parraguez VH, Hidalgo M, Saffie P. Mitochondrial distribution and meiotic progression in canine oocytes during in vivo and in vitro maturation. Theriogenology. 2011; 75:346–53. 10.1016/j.theriogenology.2010.09.005 21074834

[8] May-Panloup P, Chretien MF, Malthiery Y, Reynier P. Mitochondrial DNA in the oocyte and the developing embryo. Curr Top Dev Biol. 2007; 77:51–83. 10.1016/S0070-2153(06)77003-X 17222700

[9] Chappel S. The role of mitochondria from mature oocyte to viable blastocyst. Obstet Gynecol Int. 2013; 2013:183024. 10.1155/2013/183024 23766762 PMC3671549

[10] Iwata H, Goto H, Tanaka H, Sakaguchi Y, Kimura K, Kuwayama T, Monji Y. Effect of maternal age on mitochondrial DNA copy number, ATP content and IVF outcome of bovine oocytes. Reprod Fertil Dev. 2011; 23:424–32. 10.1071/RD10133 21426860

[11] Estienne A, Price CA. The fibroblast growth factor 8 family in the female reproductive tract. Reproduction. 2018; 155:R53–62. 10.1530/REP-17-0542 29269444

[12] Fang CX, Nong YQ, Liu FH, Fan L, Chen Y. Heparin-binding epidermal growth factor-like growth factor enhances aquaporin 3 expression and function during mouse embryo implantation. Reprod Sci. 2017; 24:463–70. 10.1177/1933719116657893 27436370

[13] Hellström A, Ley D, Hansen-Pupp I, Hallberg B, Ramenghi LA, Löfqvist C, Smith LE, Hård AL. Role of insulinlike growth factor 1 in fetal development and in the early postnatal life of premature infants. Am J Perinatol. 2016; 33:1067–71. 10.1055/s-0036-1586109 27603537 PMC5779855

[14] Li Y, Liu H, Yu Q, Liu H, Huang T, Zhao S, Ma J, Zhao H. Growth hormone promotes in vitro maturation of human oocytes. Front Endocrinol (Lausanne). 2019; 10:485. 10.3389/fendo.2019.00485 31396155 PMC6667636

[15] Baumgarten SC, Convissar SM, Zamah AM, Fierro MA, Winston NJ, Scoccia B, Stocco C. FSH regulates IGF-2 expression in human granulosa cells in an AKT-dependent manner. J Clin Endocrinol Metab. 2015; 100:E1046–55. 10.1210/jc.2015-1504 26066673 PMC4524996

[16] Giudice LC. Insulin-like growth factor family in graafian follicle development and function. J Soc Gynecol Investig. 2001; 8:S26–29. 10.1016/s1071-5576(00)00102-7 11223367

[17] Engström W, Shokrai A, Otte K, Granérus M, Gessbo A, Bierke P, Madej A, Sjölund M, Ward A. Transcriptional regulation and biological significance of the insulin like growth factor II gene. Cell Prolif. 1998; 31:173–89. 10.1111/j.1365-2184.1998.tb01196.x 9925986 PMC6647699

[18] Jiang Z, Dong H, Zheng X, Marjani SL, Donovan DM, Chen J, Tian XC. mRNA levels of imprinted genes in bovine in vivo oocytes, embryos and cross species comparisons with humans, mice and pigs. Sci Rep. 2015; 5:17898. 10.1038/srep17898 26638780 PMC4671149

[19] Livingstone C, Borai A. Insulin-like growth factor-II: its role in metabolic and endocrine disease. Clin Endocrinol (Oxf). 2014; 80:773–81. 10.1111/cen.12446 24593700

[20] Liu HB, Muhammad T, Guo Y, Li MJ, Sha QQ, Zhang CX, Liu H, Zhao SG, Zhao H, Zhang H, Du YZ, Sun K, Liu K, et al. RNA-binding protein IGF2BP2/IMP2 is a critical maternal activator in early zygotic genome activation. Adv Sci (Weinh). 2019; 6:1900295. 10.1002/advs.201900295 31406667 PMC6685478

[21] Louhio H, Hovatta O, Sjöberg J, Tuuri T. The effects of insulin, and insulin-like growth factors I and II on human ovarian follicles in long-term culture. Mol Hum Reprod. 2000; 6:694–98. 10.1093/molehr/6.8.694 10908278

[22] Sirotkin AV, Dukesová J, Makarevich AV, Kubek A, Bulla J. Evidence that growth factors IGF-I, IGF-II and EGF can stimulate nuclear maturation of porcine oocytes via intracellular protein kinase A. Reprod Nutr Dev. 2000; 40:559–69. 10.1051/rnd:2000137 11286285

[23] Harris LK, Crocker IP, Baker PN, Aplin JD, Westwood M. IGF2 actions on trophoblast in human placenta are regulated by the insulin-like growth factor 2 receptor, which can function as both a signaling and clearance receptor. Biol Reprod. 2011; 84:440–46. 10.1095/biolreprod.110.088195 20980691 PMC3043127

[24] Wang TH, Chang CL, Wu HM, Chiu YM, Chen CK, Wang HS. Insulin-like growth factor-II (IGF-II), IGF-binding protein-3 (IGFBP-3), and IGFBP-4 in follicular fluid are associated with oocyte maturation and embryo development. Fertil Steril. 2006; 86:1392–401. 10.1016/j.fertnstert.2006.03.064 17070193

[25] Highet AR, Bianco-Miotto T, Pringle KG, Peura A, Bent S, Zhang J, Nottle MB, Thompson JG, Roberts CT. A novel embryo culture media supplement that improves pregnancy rates in mice. Reproduction. 2017; 153:327–40. 10.1530/REP-16-0517 28073983

[26] Martín-Montañez E, Millon C, Boraldi F, Garcia-Guirado F, Pedraza C, Lara E, Santin LJ, Pavia J, Garcia-Fernandez M. IGF-II promotes neuroprotection and neuroplasticity recovery in a long-lasting model of oxidative damage induced by glucocorticoids. Redox Biol. 2017; 13:69–81. 10.1016/j.redox.2017.05.012 28575743 PMC5454142

[27] Pagan ML, Radhakrishnan VK, De Leon D. IGF2 regulates mitochondrial cell energy phenotype and biogenesis in TNBC cells. AACR. 2017; 77 10.1158/1538-7445.AM2017-4421

[28] Greene AD, Patounakis G, Segars JH. Genetic associations with diminished ovarian reserve: a systematic review of the literature. J Assist Reprod Genet. 2014; 31:935–46. 10.1007/s10815-014-0257-5 24840722 PMC4130940

[29] Wu X, Hu F, Zeng J, Han L, Qiu D, Wang H, Ge J, Ying X, Wang Q. NMNAT2-mediated NAD^+^ generation is essential for quality control of aged oocytes. Aging Cell. 2019; 18:e12955. 10.1111/acel.12955 30909324 PMC6516161

[30] Schatten H, Sun QY, Prather R. The impact of mitochondrial function/dysfunction on IVF and new treatment possibilities for infertility. Reprod Biol Endocrinol. 2014; 12:111. 10.1186/1477-7827-12-111 25421171 PMC4297407

[31] Pasquariello R, Ermisch AF, Silva E, McCormick S, Logsdon D, Barfield JP, Schoolcraft WB, Krisher RL. Alterations in oocyte mitochondrial number and function are related to spindle defects and occur with maternal aging in mice and humans†. Biol Reprod. 2019; 100:971–81. 10.1093/biolre/ioy248 30476005

[32] Duncan FE, Jasti S, Paulson A, Kelsh JM, Fegley B, Gerton JL. Age-associated dysregulation of protein metabolism in the mammalian oocyte. Aging Cell. 2017; 16:1381–93. 10.1111/acel.12676 28994181 PMC5676066

[33] Sha QQ, Yu JL, Guo JX, Dai XX, Jiang JC, Zhang YL, Yu C, Ji SY, Jiang Y, Zhang SY, Shen L, Ou XH, Fan HY. CNOT6L couples the selective degradation of maternal transcripts to meiotic cell cycle progression in mouse oocyte. EMBO J. 2018; 37:e99333. 10.15252/embj.201899333 30478191 PMC6293276

[34] Sugiyama M, Kawahara-Miki R, Kawana H, Shirasuna K, Kuwayama T, Iwata H. Resveratrol-induced mitochondrial synthesis and autophagy in oocytes derived from early antral follicles of aged cows. J Reprod Dev. 2015; 61:251–59. 10.1262/jrd.2015-001 25866375 PMC4547982

[35] Tsukamoto S, Kuma A, Murakami M, Kishi C, Yamamoto A, Mizushima N. Autophagy is essential for preimplantation development of mouse embryos. Science. 2008; 321:117–20. 10.1126/science.1154822 18599786

[36] Shen XH, Jin YX, Liang S, Kwon JW, Zhu JW, Lei L, Kim NH. Autophagy is required for proper meiosis of porcine oocytes maturing in vitro. Sci Rep. 2018; 8:12581. 10.1038/s41598-018-29872-y 30135500 PMC6105682

[37] Liu MJ, Sun AG, Zhao SG, Liu H, Ma SY, Li M, Huai YX, Zhao H, Liu HB. Resveratrol improves in vitro maturation of oocytes in aged mice and humans. Fertil Steril. 2018; 109:900–07. 10.1016/j.fertnstert.2018.01.020 29778389

[38] Eppig JJ. Coordination of nuclear and cytoplasmic oocyte maturation in eutherian mammals. Reprod Fertil Dev. 1996; 8:485–89. 10.1071/rd9960485 8870074

[39] Nagaoka SI, Hassold TJ, Hunt PA. Human aneuploidy: mechanisms and new insights into an age-old problem. Nat Rev Genet. 2012; 13:493–504. 10.1038/nrg3245 22705668 PMC3551553

[40] Tkachenko O, Ting A, Xu J, Stouffer R. Insulin-like growth factor-2 (IGF2) production and regulation in macaque preantral follicles during 3-dimensional culture. Fertil Steril. 2016; 106:e119 10.1016/j.fertnstert.2016.07.357

[41] Sferruzzi-Perri AN. Regulating needs: exploring the role of insulin-like growth factor-2 signalling in materno-fetal resource allocation. Placenta. 2018 (Suppl 1); 64:S16–22. 10.1016/j.placenta.2018.01.005 29352601

[42] Ceda GP, Dall’Aglio E, Magnacavallo A, Vargas N, Fontana V, Maggio M, Valenti G, Lee PD, Hintz RL, Hoffman AR. The insulin-like growth factor axis and plasma lipid levels in the elderly. J Clin Endocrinol Metab. 1998; 83:499–502. 10.1210/jcem.83.2.4548 9467564

[43] Cline BH, Steinbusch HW, Malin D, Revishchin AV, Pavlova GV, Cespuglio R, Strekalova T. The neuronal insulin sensitizer dicholine succinate reduces stress-induced depressive traits and memory deficit: possible role of insulin-like growth factor 2. BMC Neurosci. 2012; 13:110. 10.1186/1471-2202-13-110 22989159 PMC3564824

[44] Andrus BM, Blizinsky K, Vedell PT, Dennis K, Shukla PK, Schaffer DJ, Radulovic J, Churchill GA, Redei EE. Gene expression patterns in the hippocampus and amygdala of endogenous depression and chronic stress models. Mol Psychiatry. 2012; 17:49–61. 10.1038/mp.2010.119 21079605 PMC3117129

[45] Louvi A, Accili D, Efstratiadis A. Growth-promoting interaction of IGF-II with the insulin receptor during mouse embryonic development. Dev Biol. 1997; 189:33–48. 10.1006/dbio.1997.8666 9281335

[46] Rappolee DA, Sturm KS, Behrendtsen O, Schultz GA, Pedersen RA, Werb Z. Insulin-like growth factor II acts through an endogenous growth pathway regulated by imprinting in early mouse embryos. Genes Dev. 1992; 6:939–52. 10.1101/gad.6.6.939 1317321

[47] Frasca F, Pandini G, Scalia P, Sciacca L, Mineo R, Costantino A, Goldfine ID, Belfiore A, Vigneri R. Insulin receptor isoform A, a newly recognized, high-affinity insulin-like growth factor II receptor in fetal and cancer cells. Mol Cell Biol. 1999; 19:3278–88. 10.1128/mcb.19.5.3278 10207053 PMC84122

[48] Neirijnck Y, Papaioannou MD, Nef S. The insulin/IGF system in mammalian sexual development and reproduction. Int J Mol Sci. 2019; 20:4440. 10.3390/ijms20184440 31505893 PMC6770468

[49] Choi WJ, Banerjee J, Falcone T, Bena J, Agarwal A, Sharma RK. Oxidative stress and tumor necrosis factor-alpha-induced alterations in metaphase II mouse oocyte spindle structure. Fertil Steril. 2007; 88:1220–31. 10.1016/j.fertnstert.2007.02.067 17601599

[50] Zhang X, Wu XQ, Lu S, Guo YL, Ma X. Deficit of mitochondria-derived ATP during oxidative stress impairs mouse MII oocyte spindles. Cell Res. 2006; 16:841–50. 10.1038/sj.cr.7310095 16983401

[51] Ben-Meir A, Burstein E, Borrego-Alvarez A, Chong J, Wong E, Yavorska T, Naranian T, Chi M, Wang Y, Bentov Y, Alexis J, Meriano J, Sung HK, et al. Coenzyme Q10 restores oocyte mitochondrial function and fertility during reproductive aging. Aging Cell. 2015; 14:887–95. 10.1111/acel.12368 26111777 PMC4568976

[52] Takahashi T, Takahashi E, Igarashi H, Tezuka N, Kurachi H. Impact of oxidative stress in aged mouse oocytes on calcium oscillations at fertilization. Mol Reprod Dev. 2003; 66:143–52. 10.1002/mrd.10341 12950101

[53] Susor A, Jansova D, Cerna R, Danylevska A, Anger M, Toralova T, Malik R, Supolikova J, Cook MS, Oh JS, Kubelka M. Temporal and spatial regulation of translation in the mammalian oocyte via the mTOR-eIF4F pathway. Nat Commun. 2015; 6:6078. 10.1038/ncomms7078 25629602 PMC4317492

[54] Jansova D, Tetkova A, Koncicka M, Kubelka M, Susor A. Localization of RNA and translation in the mammalian oocyte and embryo. PLoS One. 2018; 13:e0192544. 10.1371/journal.pone.0192544 29529035 PMC5846722

[55] Teerink H, Kasperaitis MA, De Moor CH, Voorma HO, Thomas AA. Translation initiation on the insulin-like growth factor II leader 1 is developmentally regulated. Biochem J. 1994; 303:547–53. 10.1042/bj3030547 7980416 PMC1137362

[56] Nasheed Hamad Almohammed Z, Moghani-Ghoroghi F, Ragerdi-Kashani I, Fathi R, Tahaei LS, Naji M, Pasbakhsh P. The effect of melatonin on mitochondrial function and autophagy in in vitro matured oocytes of aged mice. Cell J. 2020; 22:9–16. 10.22074/cellj.2020.6302 31606961 PMC6791077

[57] Wang Y, Gan G, Wang B, Wu J, Cao Y, Zhu D, Xu Y, Wang X, Han H, Li X, Ye M, Zhao J, Mi J. Cancer-associated fibroblasts promote irradiated cancer cell recovery through autophagy. EBioMedicine. 2017; 17:45–56. 10.1016/j.ebiom.2017.02.019 28258923 PMC5360585

[58] Shimizu T, Sugihara E, Yamaguchi-Iwai S, Tamaki S, Koyama Y, Kamel W, Ueki A, Ishikawa T, Chiyoda T, Osuka S, Onishi N, Ikeda H, Kamei J, et al. IGF2 preserves osteosarcoma cell survival by creating an autophagic state of dormancy that protects cells against chemotherapeutic stress. Cancer Res. 2014; 74:6531–41. 10.1158/0008-5472.CAN-14-0914 25273088

[59] Tatone C, Carbone MC, Gallo R, Delle Monache S, Di Cola M, Alesse E, Amicarelli F. Age-associated changes in mouse oocytes during postovulatory in vitro culture: possible role for meiotic kinases and survival factor BCL2. Biol Reprod. 2006; 74:395–402. 10.1095/biolreprod.105.046169 16251501

[60] Wu J, Zhang L, Wang X. Maturation and apoptosis of human oocytes in vitro are age-related. Fertil Steril. 2000; 74:1137–41. 10.1016/s0015-0282(00)01597-1 11119740

[61] Perez GI, Tilly JL. Cumulus cells are required for the increased apoptotic potential in oocytes of aged mice. Hum Reprod. 1997; 12:2781–83. 10.1093/humrep/12.12.2781 9455852

[62] Chu N, Gui Y, Qiu X, Zhang N, Li L, Li D, Tang W, Gober HJ, Zhang B, Wang L. The effect of DHEA on apoptosis and cohesin levels in oocytes in aged mice. Biosci Trends. 2017; 11:427–38. 10.5582/bst.2017.01108 28717062

[63] Sferruzzi-Perri AN, Sandovici I, Constancia M, Fowden AL. Placental phenotype and the insulin-like growth factors: resource allocation to fetal growth. J Physiol. 2017; 595:5057–93. 10.1113/JP273330 28337745 PMC5538190

[64] Li Y, Liu H, Wu K, Liu H, Huang T, Chen ZJ, Zhao S, Ma J, Zhao H. Melatonin promotes human oocyte maturation and early embryo development by enhancing clathrin-mediated endocytosis. J Pineal Res. 2019; 67:e12601. 10.1111/jpi.12601 31361919

