# Supplementary material for: IGF2 improves the developmental competency and meiotic structure of oocytes from aged mice
Source: Aging (Albany NY). 2020 Dec 9;13(2):2118–34. doi: 10.18632/aging.202214 (PMC7880328; doi:10.18632/aging.202214)
Supplement: Supplementary Table 1 [file aging-13-202214-s001.pdf]

SUPPLEMENTARY TABLE

Supplementary Table 1. Primer sequences for qRT-PCR.

	Forward	Reverse
IGF22	TTCTACTTCAGCAGGCCTTCAA	ATATTGGAAGAACTTGCCCACG
SIRT1	CTGTTGACCGATGGACTCCT	GCCACAGCGTCATATCATCC
BMP15	TCCTTGCTGACGACCCTACAT	TACCTCAGGGGATAGCCTTGG
GDF9	TCTTAGTAGCCTTAGCTCTCAGG	TGTCAGTCCCATCTACAGGCA
SOD1	GCTGTACCAGTGCAGGTCCTCA	CATTTCCACCTTTGCCCAAGTC
